# Supplementary material for: Neural and behavioral signatures of the multidimensionality of manipulable object processing
Source: Commun Biol. 2023 Sep 14;6:940. doi: 10.1038/s42003-023-05323-x (PMC10502059; doi:10.1038/s42003-023-05323-x)

SUPPLEMENTARY MATERIAL FOR

Neural and behavioral signatures of the multidimensionality of object processing.

Jorge Almeida[1,2*], Alessio Fracasso[3], Stephanie Kristensen[1,2], Daniela Valério[1,2], Fredrik Bergström[1,2,4], Ramakrishna Chakravarthi[5], Zohar Tal[1,2], Jonathan Walbrin[1,2]

1. Proaction Lab, Faculty of Psychology and Educational Sciences, University of Coimbra, Portugal.

2. CINEICC, Faculty of Psychology and Educational Sciences, University of Coimbra, Portugal.

3. School of Psychology and Neuroscience, University of Glasgow, UK

4. Department of Psychology, University of Gothenburg, Sweden

5. School of Psychology, University of Aberdeen, UK

*Corresponding Author:

Jorge Almeida

Faculty of Psychology and Educational Sciences

University of Coimbra

jorgecbalmeida@gmail.com

This section includes two supplementary tables and five supplementary figures:

Table S1. Items used in all of the experiments.

Figure S1. Examples of the 80 selected manipulable objects.

Figure S2. Stress values for the non-metric MDS analysis per each knowledge type.

Figure S3. Labels generated per dimension.

Figure S4. Neural effects of the object-related dimensions against 0 (i.e., against no modulation).

Figure S5. Between-dimension correlations.

Table S1. Items used in all of the experiments.

Portuguese	English	Portuguese	English
abre caricas	bottle opener	fósforo	match
afia lápis	pencil sharpener	furador	hole puncher
agrafador	Stapler	garfo	fork
agulha	needle	garrafa	bottle
alicate	pliers	guardanapo	napkin
apagador	board eraser	isqueiro	lighter
apito	whistle	jarro	pitcher
balde	bucket	lanterna	flashlight
batedeira manual	whisk	lápis	pencil
berbequim	electric drill	leque	(handheld) fan
boia	buoy/floater	lima das unhas	nail file
bola de basquete	basketball	lupa	magnifier
borracha	eraser	manípulo da porta	door handle
borrifador	spray bottle	máquina de barbear	electric shaver
broca	drill bit	martelo	hammer
buzina	horn	moedor de pimenta	pepper grinder
cabide	clothes hanger	mola da roupa	clothes pin
cana de pesca	fishing pole	pá	shovel
canivete	swiss army knife	parafuso	screw
carimbo	stamp	peso	dumbbell
carrinho de compras	shopping cart	pião	spinning top
castiçal	candle holder	pinça	tweezers
chave	key	pincel	paint brush
chave inglesa	wrench	prego	nail
chávena	cup	quebra nozes	nut cracker
clip	paper clip	ralador	grater
colher	spoon	raquete	tennis racket
colher de pau	wooden spoon	rato computador	computer mouse
copo	glass	remo	oar
corta-unhas	nail clipper	rolha	cork
dardo	dart	rolo da massa	rolling pin
descascador	peeler	saco de pasteleiro	pastry bag
desentupidor	plunger	secador	hair dryer
enxada	hoe	seringa	syringe
escova de cabelo	hair brush	taco de golfe	golf club
escova de dentes	toothbrush	tampa de garrafa	bottle cap
esfregona	mop	tesoura	scissors
esponja	(kitchen) sponge	tigela	bowl
espremedor	citrus squeezer	varinha mágica	handheld blender
faca	knife	vassoura	broom

Here we present the original items used (in Portuguese) as well as the English translation of the Portuguese words used.

Figure S1. Examples of the 80 selected manipulable objects. Here, we present one of the ten exemplars for each of the 80 manipulable objects used in Experiment 4.

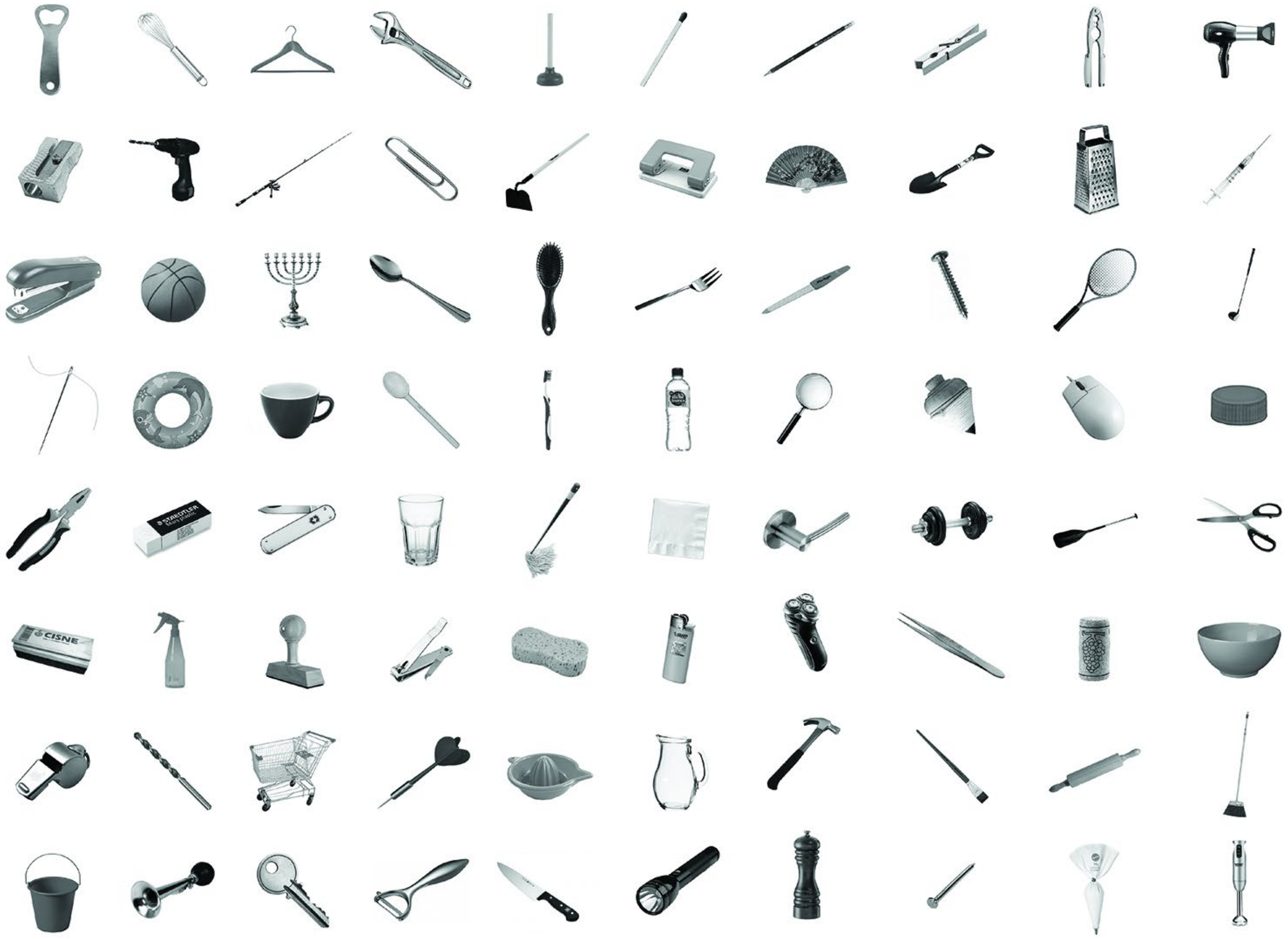

Figure S2. Stress values for the non-metric MDS analysis per each knowledge type. Here we show Kruskal Stress values for the different dimensional solutions for **A)** Function; **B)** Manipulation; and **C)** Vision. We chose the solutions with a stress value right below 0.1.

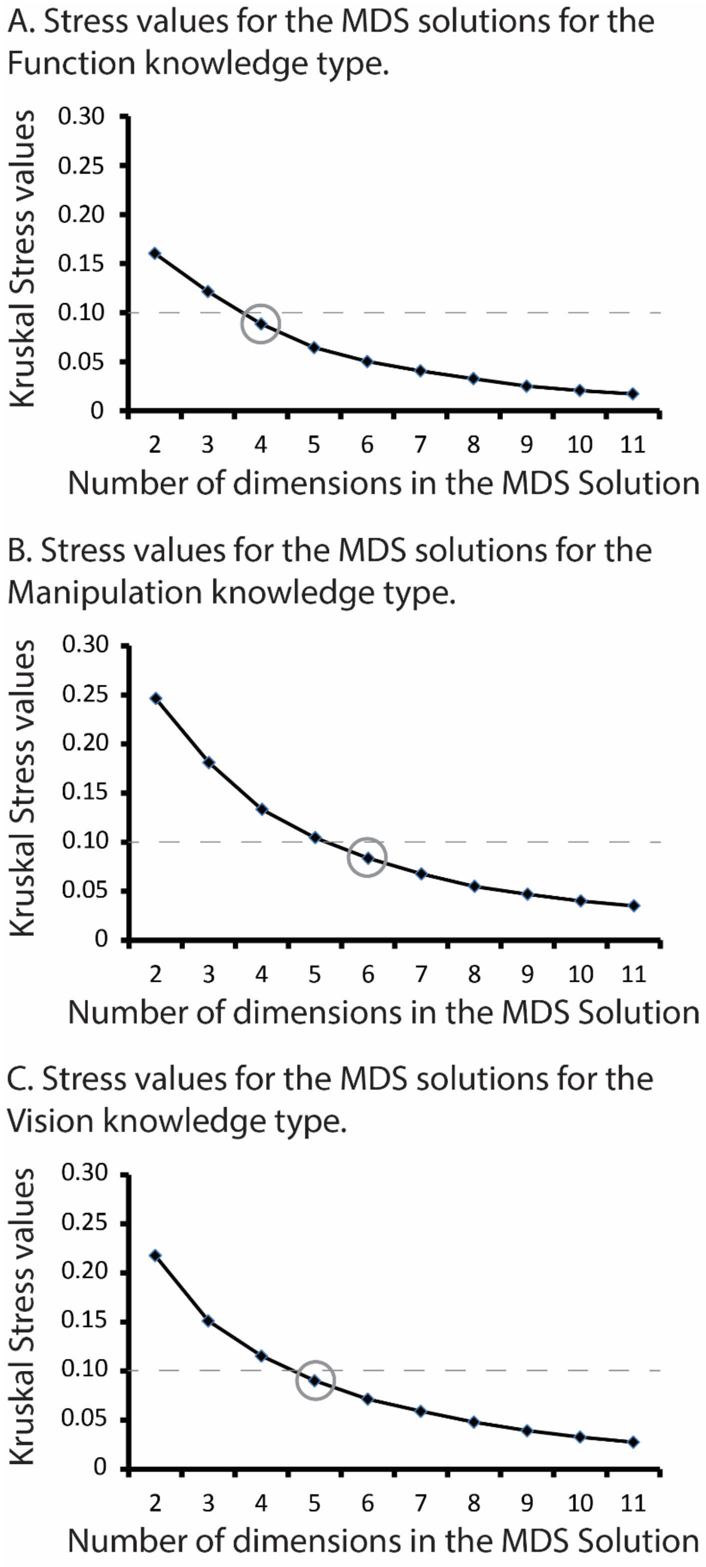

Figure S3. Labels generated per dimension. Here, we show percent label generation per dimension, along with an example of the images presented to the participant, for A) function; B) Manipulation; and C) Vision. Labels presented are collated from individual labels generated. Bars in green correspond to the labels that were put together as the winning label.

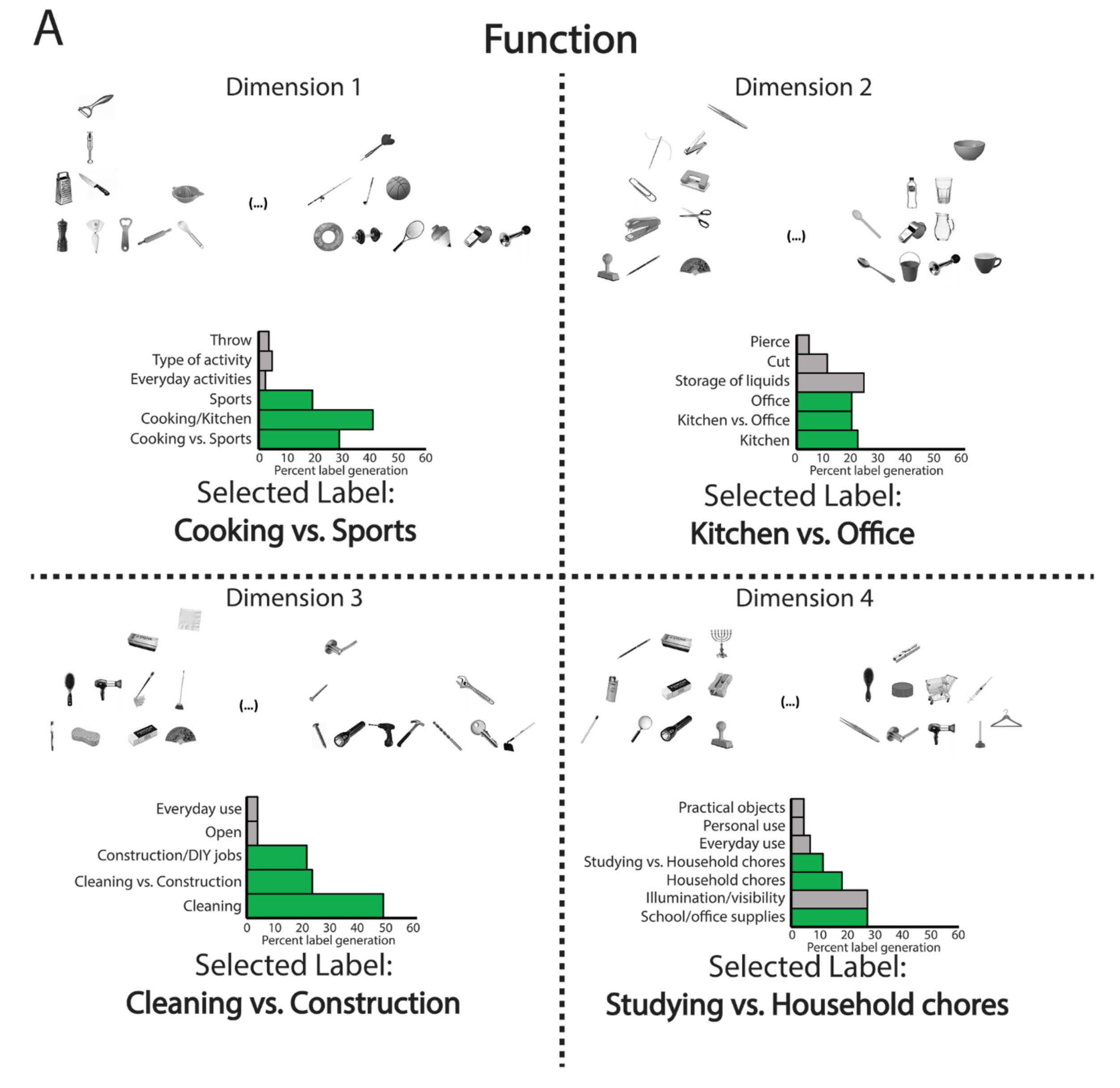

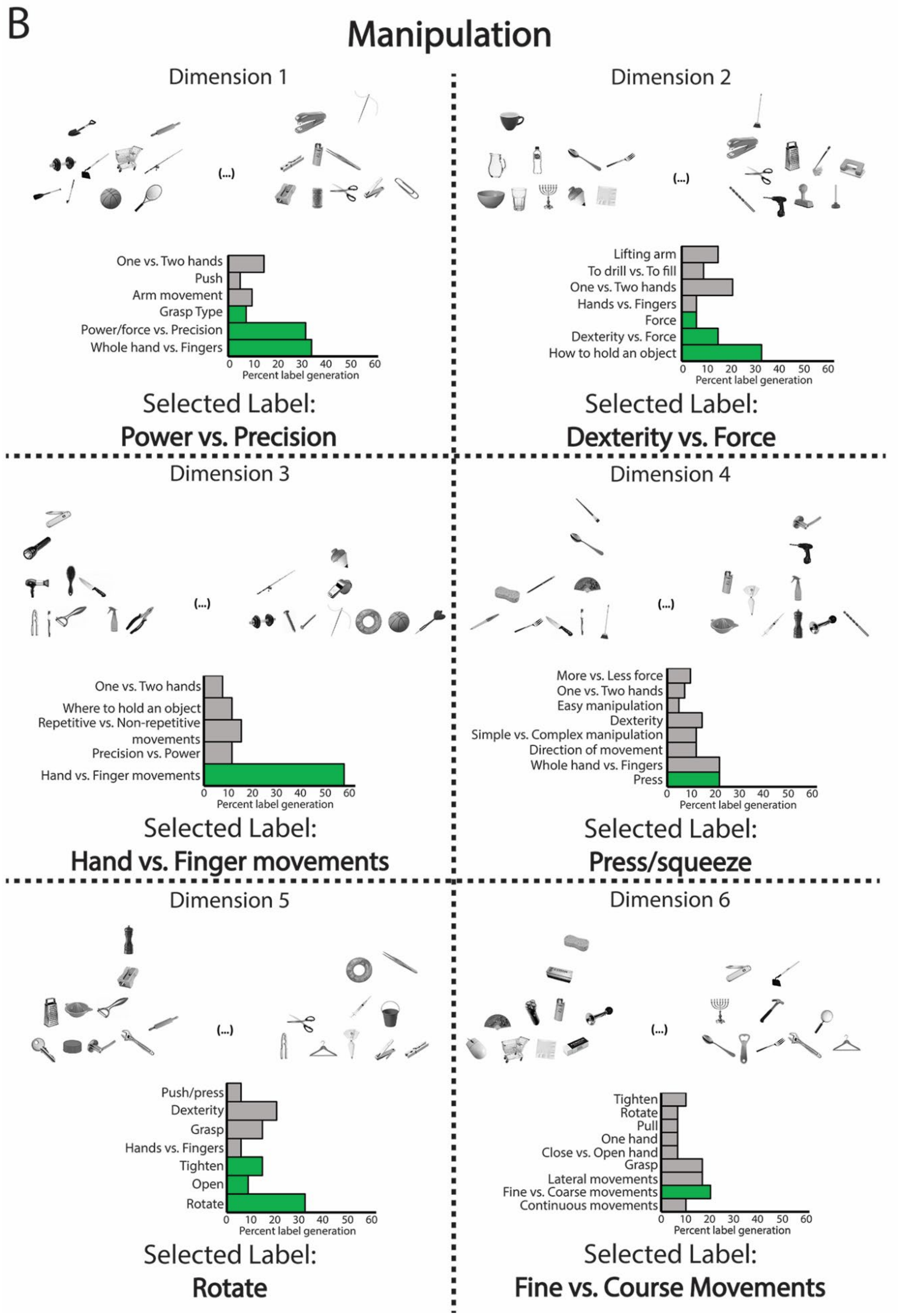
B
Manipulation
Dimension 1
(...)
One vs. Two hands
Push
Arm movement
Grasp Type
Power/force vs. Precision
Whole hand vs. Fingers
0 10 20 30 40 50 60
Percent label generation
Selected Label:
Power vs. Precision
Dimension 2
(...)
Lifting arm
To drill vs. To fill
One vs. Two hands
Hands vs. Fingers
Force
Dexterity vs. Force
How to hold an object
0 10 20 30 40 50 60
Percent label generation
Selected Label:
Dexterity vs. Force
Dimension 3
(...)
One vs. Two hands
Where to hold an object
Repetitive vs. Non-repetitive movements
Precision vs. Power
Hand vs. Finger movements
0 10 20 30 40 50 60
Percent label generation
Selected Label:
Hand vs. Finger movements
Dimension 4
(...)
More vs. Less force
One vs. Two hands
Easy manipulation
Dexterity
Simple vs. Complex manipulation
Direction of movement
Whole hand vs. Fingers
Press
0 10 20 30 40 50 60
Percent label generation
Selected Label:
Press/squeeze
Dimension 5
(...)
Push/press
Dexterity
Grasp
Hands vs. Fingers
Tighten
Open
Rotate
0 10 20 30 40 50 60
Percent label generation
Selected Label:
Rotate
Dimension 6
(...)
Tighten
Rotate
Pull
One hand
Close vs. Open hand
Grasp
Lateral movements
Fine vs. Coarse movements
Continuous movements
0 10 20 30 40 50 60
Percent label generation
Selected Label:
Fine vs. Course Movements

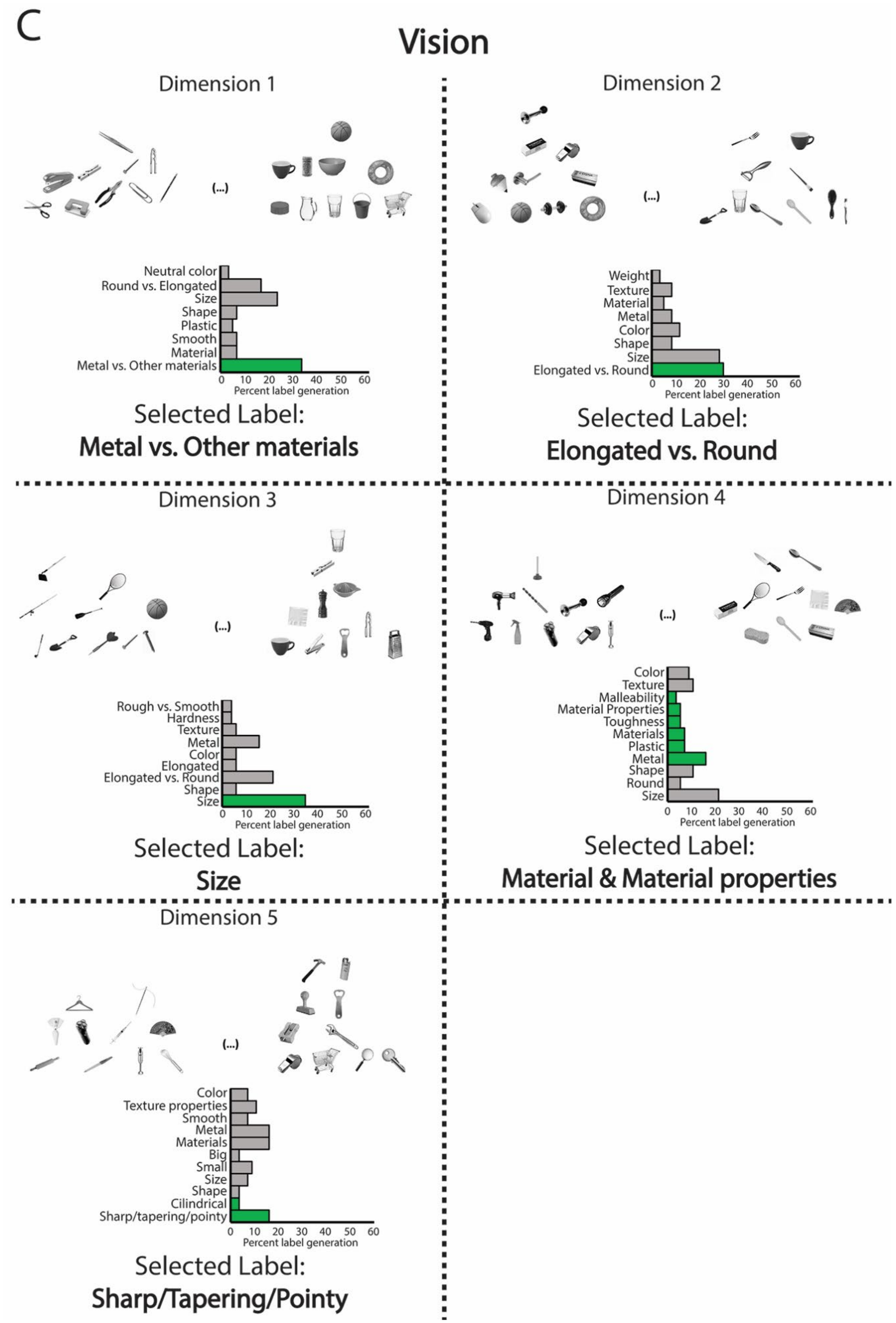
C
Vision
Dimension 1
(...)
Neutral color
Round vs. Elongated
Size
Shape
Plastic
Smooth
Material
Metal vs. Other materials
0 10 20 30 40 50 60
Percent label generation
Selected Label:
Metal vs. Other materials
Dimension 2
(...)
Weight
Texture
Material
Metal
Color
Shape
Size
Elongated vs. Round
0 10 20 30 40 50 60
Percent label generation
Selected Label:
Elongated vs. Round
Dimension 3
(...)
Rough vs. Smooth
Hardness
Texture
Metal
Color
Elongated
Elongated vs. Round
Shape
Size
0 10 20 30 40 50 60
Percent label generation
Selected Label:
Size
Dimension 4
(...)
Color
Texture
Malleability
Material Properties
Toughness
Materials
Plastic
Metal
Shape
Round
Size
0 10 20 30 40 50 60
Percent label generation
Selected Label:
Material & Material properties
Dimension 5
(...)
Color
Texture properties
Smooth
Metal
Materials
Big
Small
Size
Shape
Cilindrical
Sharp/tapering/pointy
0 10 20 30 40 50 60
Percent label generation
Selected Label:
Sharp/Tapering/Pointy

Figure S4. Neural effects of the object-related dimensions against 0 (i.e., against no modulation). Here we show dimension-specific F-maps against 0 for A) Function; B) Manipulation; and C) Vision. In D), we also show F-maps for "Cooking vs. Sports" cluster-forming height-thresholded at p<.001 and corrected at FDR p<.05. No cluster survived at this cluster-forming threshold for the dimension "Material properties".

A

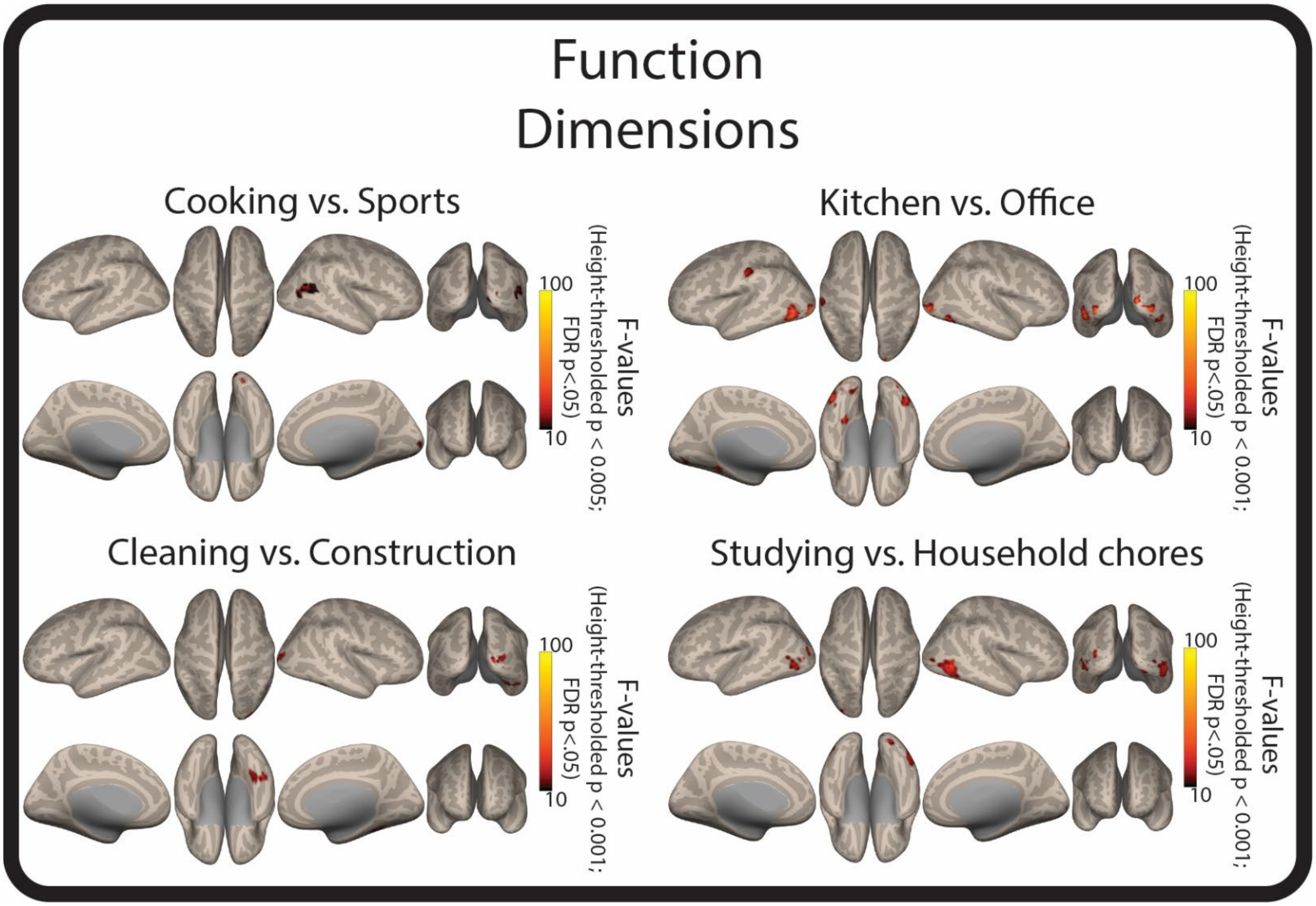

B

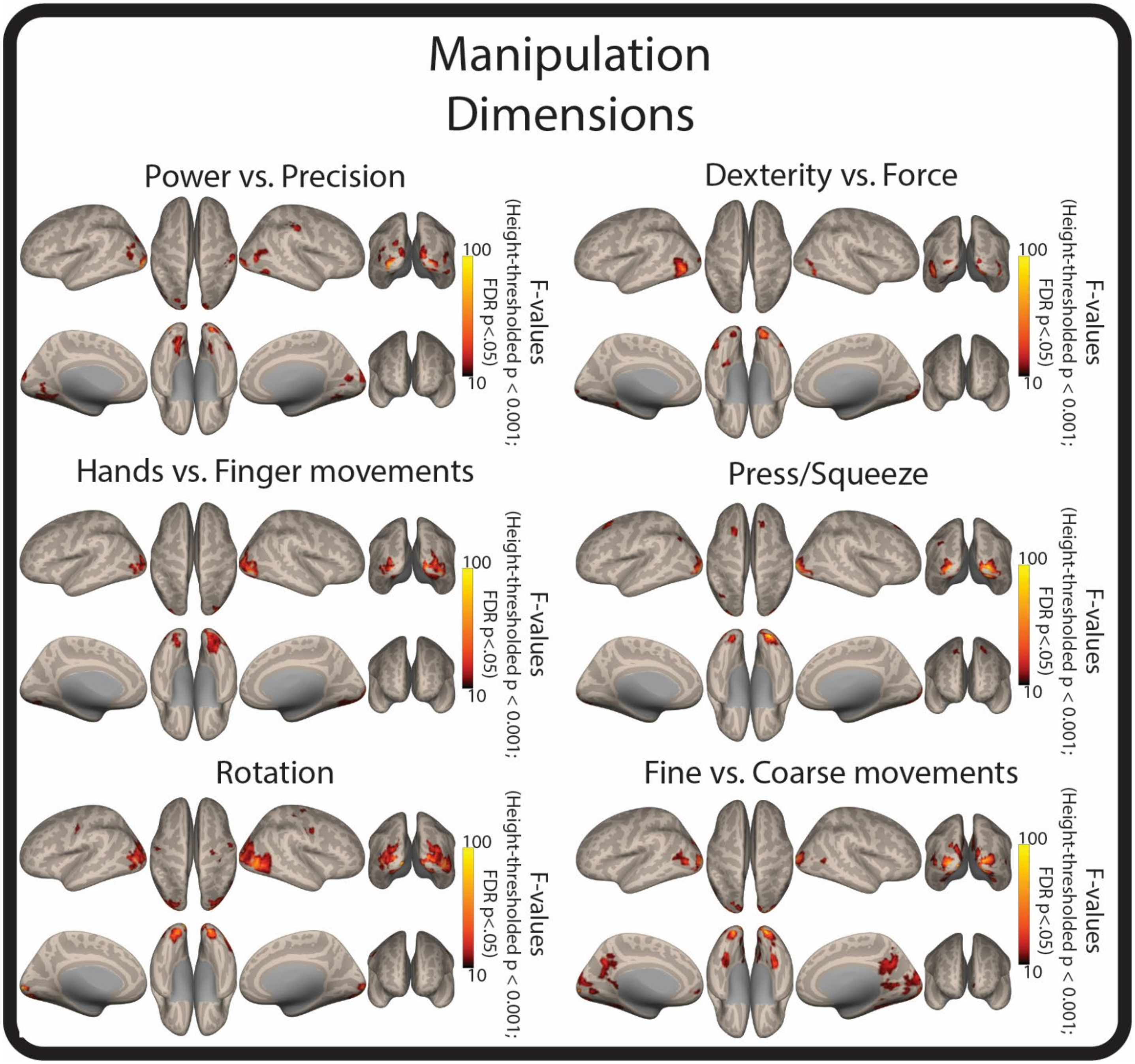
Manipulation Dimensions
Power vs. Precision
Dexterity vs. Force
Hands vs. Finger movements
Press/Squeeze
Rotation
Fine vs. Coarse movements
F-values
(Height-thresholded p < 0.001; FDR p<.05)
100
10

C

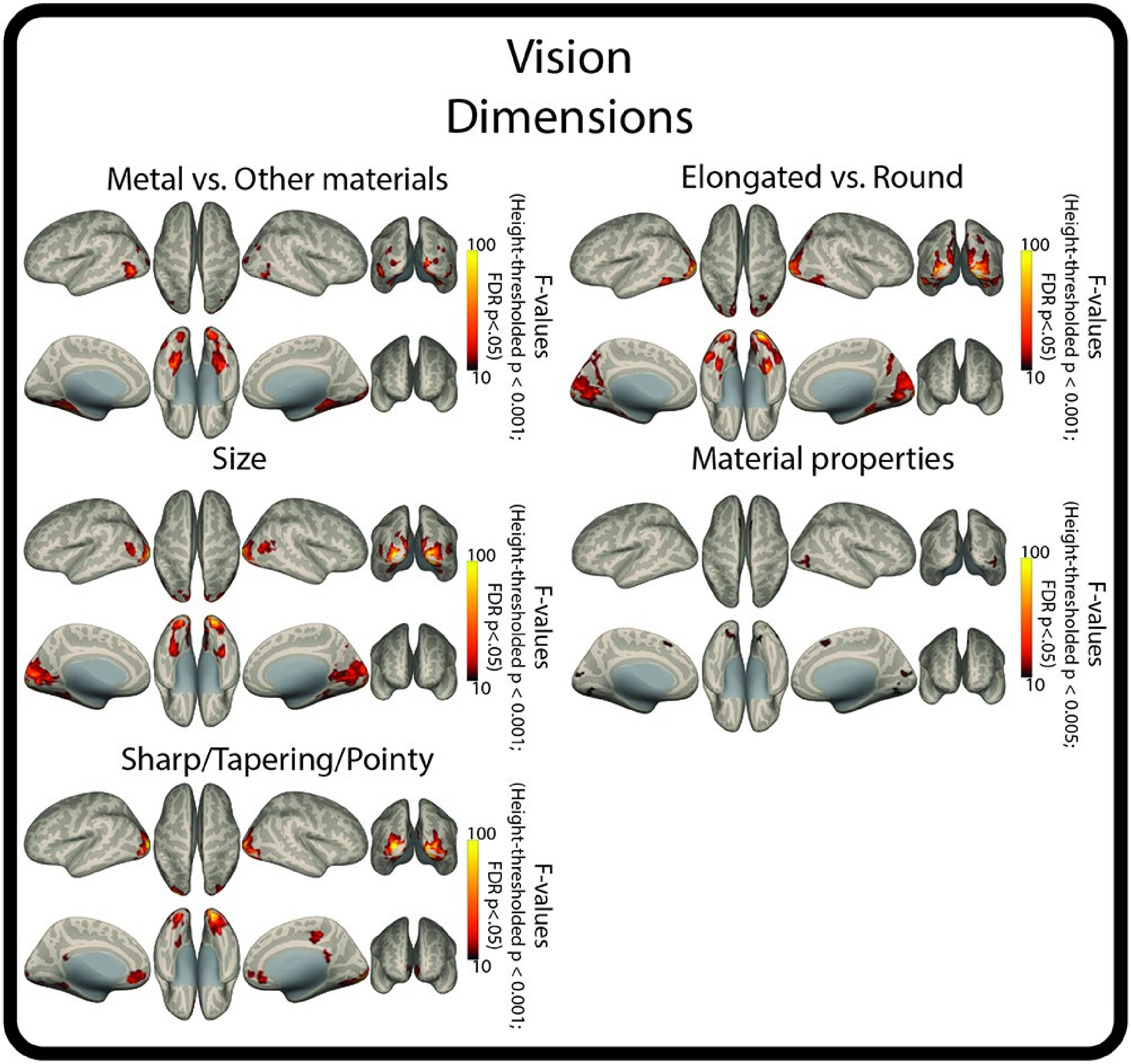

Vision
Dimensions
Metal vs. Other materials
F-values
(Height-thresholded p < 0.001;
FDR p<.05)
100
10
Elongated vs. Round
F-values
(Height-thresholded p < 0.001;
FDR p<.05)
100
10
Size
F-values
(Height-thresholded p < 0.001;
FDR p<.05)
100
10
Material properties
F-values
(Height-thresholded p < 0.005;
FDR p<.05)
100
10
Sharp/Tapering/Pointy
F-values
(Height-thresholded p < 0.001;
FDR p<.05)
100
10

D

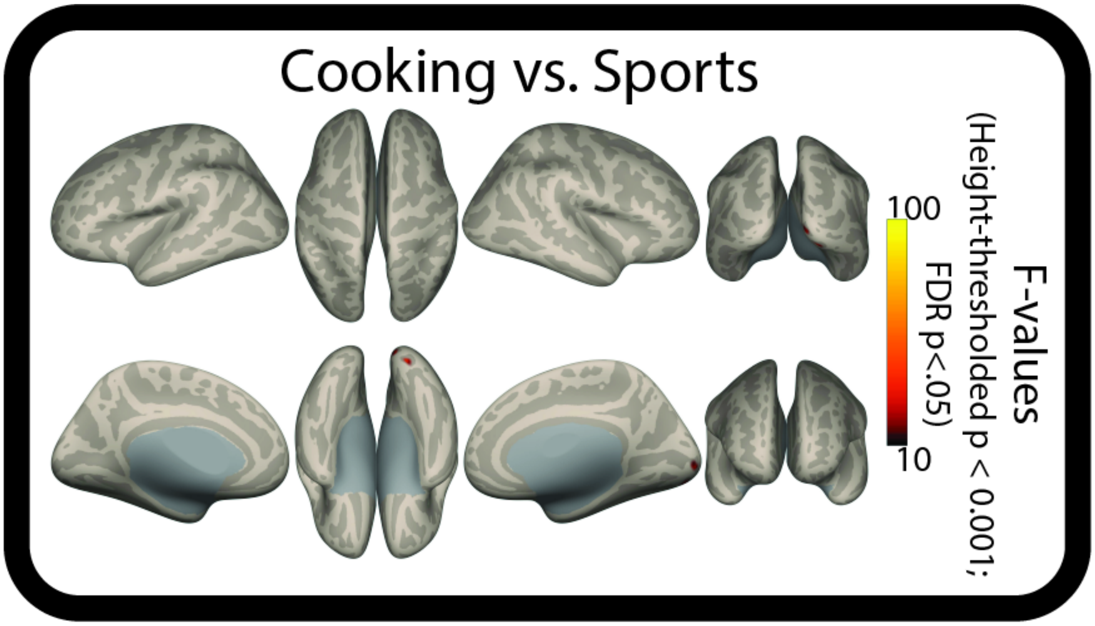
Cooking vs. Sports
F-values
(Height-thresholded p < 0.001;
FDR p<.05)
100
10

Figure S5. Between-dimension correlations. Here we show between-dimension correlations within and between knowledge types. As can be seen, there are some correlations between the dimensions that are below or above 0. Most of these are, however, small to moderate. There are 8 correlations (out of the possible 74 between knowledge-type correlations between dimensions) that are above .5, all but one below .7 (maximum is - .71: between “Metal vs. Other materials” and “Kitchen vs. Office”). Overall, then, correlations are rather low, with few exceptions. These exceptions may be expected – many of the kitchen utensils shown do have a metal part – and are part of the natural distribution of object properties in the world.

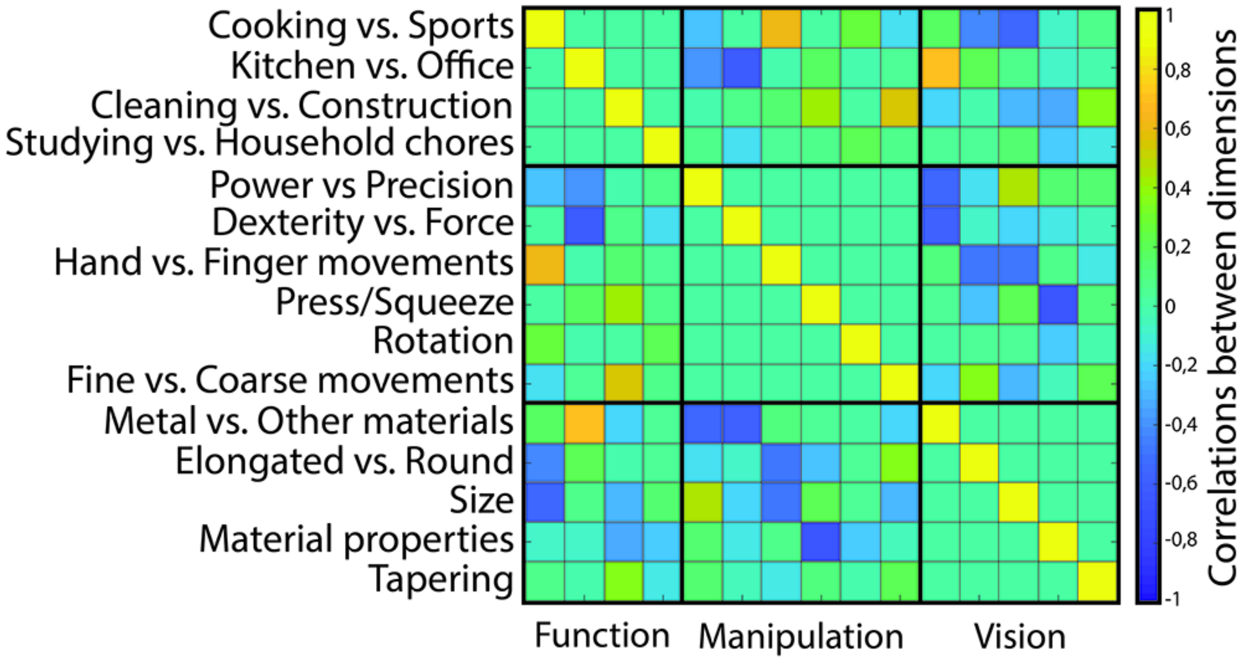

Supplement: Supplementary file 2 — Supplementary Information [file 42003_2023_5323_MOESM2_ESM.pdf]
